# The microarchitectural variability in the echinoid skeleton: a 3D geometrical and stiffness characterization of *Paracentrotus lividus*

**DOI:** 10.1098/rsos.241439

**Published:** 2025-06-25

**Authors:** Valentina Perricone, Pasquale Cesarano, Mainak Deb, Derek Lublin, Mirko Mutalipassi, Lucia Pappalardo, David Kisailus, Francesco Marmo

**Affiliations:** ^1^ Department of Materials Science and Engineering, University of California Irvine, Irvine, CA, USA; ^2^ Department of Structures for Engineering and Architecture, University of Naples Federico II, Napoli, Italy; ^3^ Amrita Vishwa Vidyapeetham, Coimbatore, Tamil Nadu, India; ^4^ Department of Integrative Marine Ecology, Stazione Zoologica Anton Dohrn, Napoli, Campania, Italy; ^5^ Istituto Nazionale di Geofisica e Vulcanologia Sezione di Napoli, Napoli, Campania, Italy; ^6^ Dipartimento di Strutture per l'Ingegneria e l'Architettura, Universita degli Studi di Napoli Federico II, Napoli, Italy

**Keywords:** sea urchin, microarchitecture, stereom, stiffness, fabric tensor

## Abstract

The sea urchin skeleton is a lightweight yet load-bearing hierarchical structure composed of calcitic plates with a species-specific three-dimensional (3D) trabecular meshwork known as stereom. Interestingly, the stereom architecture is extremely complex and variable in different basic types, each one characterized by a unique geometry and structural behaviours. The present study provides an in-depth analysis of the microarchitectural variability in the sea urchin *Paracentrotus lividus*. Accordingly, micro-CT scans, image analysis, 3D modelling, mean intercept length and linear elastic finite-element analysis were conducted to provide the first comprehensive insights on the structural variability of the different stereom types, their anisotropy and their mechanical behaviour calculated for tensile and shear loading. The findings demonstrate distinct structural adaptations, with anisotropic stereoms specializing in directional stress transfer and more isotropic stereoms facilitating a uniform stress distribution. These results provide critical insights into the mechanical functions of stereom variability and support bioinspired designs for lightweight and strong materials.

## 1. Introduction

**Figure 1.**
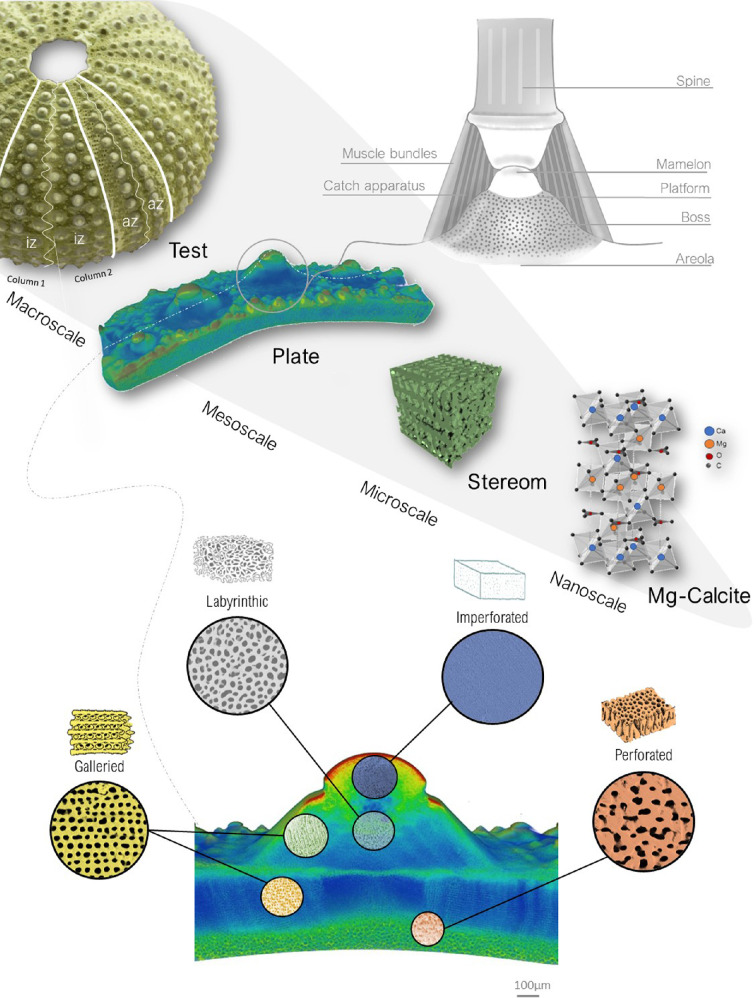
The hierarchical design of the skeleton of *Paracentrotus lividus*. At the macroscale, the skeleton exhibits a pentameric symmetry with a tessellated test, composed of alternating ambulacral (az) and interambulacral (iz) zones arranged in double columns. At the mesoscale, individual skeletal plates feature rounded tubercles, which serve as attachment sites for spines via soft tissues, including muscles and the catch apparatus. The spine–tubercle system consists of the mamelon, platform, boss and areola. At the microscale, the structure is composed of stereom, a highly porous biomineralized network. At the nanoscale, the skeletal material is primarily Mg-calcite. The bottom panel presents a longitudinal section from a micro-CT scan, illustrating stereom variability: galleried stereom at the tubercle boss and suture areas, labyrinthic stereom in the central region of the plate and perforated stereom at the basal zone.

Biological materials, despite being composed of basic components like silicates, carbonates and phosphates, exhibit extraordinary mechanical properties such as load-bearing capabilities, impact absorption and crack deflection [1–4]. These remarkable features arise from their hierarchical design, where shapes, structures and compositions are intricately organized to produce high-performance multifunctional emergent properties, such as high strength and fracture toughness [4–7]. A notable example is the hierarchical and tessellated design of the echinoid skeleton, which has revealed extensive potential for bioinspired applications in various technical fields (see [8] and literature cited therein). Echinoids, commonly known as sea urchins, are marine invertebrates belonging to the phylum Echinodermata. In order to withstand biotic (e.g. predatory attacks) and abiotic mechanical stresses (e.g. environmental forces such as fluid flow, pressure, locomotion), they have evolved a lightweight yet mechanically resilient endoskeleton with a distinctive multiscale architectural design, as summarized in electronic supplementary material, table S1 [8–11]. In particular, at the macroscale, this skeleton consists of a dome-shaped and tessellated shell structure, defined as a ‘test’ and composed of centimetre-sized polygonal skeletal plates [10,12,13] (figure 1). These plates exhibit a wide range of morphologies, each closely associated with specific functional roles [14], consisting of integrated inorganic and organic components. The inorganic component is composed of magnesium-rich calcite [15], formed via the crystallization of an amorphous calcium carbonate (ACC) precursor phase [16–19]. The Mg-calcite crystals are arranged into a porous light-weight structure, known as stereom, which is extremely variable in density and microarchitecture [8,9,14,20] (figure 1). The organic component (stroma), which fills and permeates the inorganic stereom, is a true connective tissue consisting of cells, extracellular matrix (ECM) and collagen fibril bundles [14,21]. The stroma significantly contributes to the integrity of the skeletal structure by providing mechanical strength and flexibility [22]. Based on their skeletal morphology and lifestyle, echinoids are divided in two groups: regular, which are characterized by a radial symmetry, herbivorous/carnivorous and epifaunal (i.e. living on the ocean bottom surface) lifestyle; irregular, having a bilateral symmetry and tending to be deposit feeder and infaunal (i.e. living buried in the ocean bottom) [23]. In regular echinoids, the plates are systematically arranged in a pentaradial symmetry across the test. The arrangement includes 10 double vertical columns, which represent the alternating five ambulacral zones and five interambulacral zones, each consisting of 20 plates per column [23] (figure 1A). The plates of the ambulacral zones are typically pierced by pores (two series) for the tube-feet protrusion; these pore-pairs are located and aligned along the outer (adambulacral) margins of the plates. Since all plates bear movable spines, their outer surfaces display rounded tubercles on which the spines are articulated by ball-and-socket joints. The tubercles are constructed by a ball-like ‘mamelon’ at the centre, where the spine is articulated and usually imperforated (figure 1B) [14]. The mamelon is situated at the apex of the ‘boss’, a conical mound representing the attachment site for the connective tissue, generally composed of galleried stereom [14]. The ‘areole’ is the area surrounding the base of the boss and is composed of a fine labyrinthic stereom [14]. The spine attaches to the tubercle by an inner ring of collagenous fibres, called the ‘catch apparatus’, and an outer ring of muscle fibres (generally described as ‘smooth’) [24–26]. Catch apparatus fibres are inserted within the boss and bound around the trabeculae. Muscle fibres are attached to the areole stereom through connective tendons. Finally, a fluid-filled space separates the muscle fibres from the catch apparatus [27]. At the microscale, the plate displays a complex three-dimensional (3D) stereomic architectural design [28–30]. Some of these architectures can be described as periodic minimal surfaces [31,32], usually achieving high strength and improved damage tolerance [33]. Based on Smith’s classification [14], 10 stereom types exist in different combinations to create the species-specific 3D structural patterns within the plates. These stereom types include the imperforate, microperforate, simple perforate, galleried, rectilinear, retiform, laminar, fascicular, labyrinthic and irregular perforate. Prior research has primarily focused on descriptive analyses [14,34,35], while the structural variability of these stereoms and the resulting mechanical properties that emerge from their combinations remain largely unexplored, especially in skeletal plates. While different mechanical aspects have been investigated in the stereom of other skeletal components—such as spines [19,35–41], Aristotle’s lantern [42] and teeth [18,43]—studies on the test provided only partial insights. Interesting available literatures on the test micromechanics have been provided by Müter *et al.* [44], on *Echinocardium cordatum*, Ji *et al.* [45] on *Phyllacanthus imperialis* and Yu *et al.* [46] on *Anthocidaris crassispina* and *Tripnenstes gratilla* reporting a 3D evaluation of the energy absorption behaviour of specific stereom regions (e.g. the periodic stereom of the tubercle). However, a comparative study across different stereom types within skeletal plates, as well as their species-specific 3D variability, is still lacking. This gap leaves the mechanics of stereom incompletely understood. In *Paracentrotus lividus* (Lamarck, 1816), a common regular echinoid of the Mediterranean Sea, four types of stereoms can be detected: imperforate, galleried, labyrinthic and perforate (figure 1). This specific variability has been described by Smith [14] and two-dimensional (2D) analysed using finite-element analysis (FEA) [47]. While these studies provide valuable insights, they fail to capture the full complexity of the stereom 3D geometrical variability and mechanical behaviour.

In this framework, the present study aims to gain in-depth insights about the elastic characterization of the three-dimensional (3D) microarchitectural variability within *P. lividus* test. Actually, the principal role of stereom is to bear forces and transfer stresses within the test, furnishing specific elastic properties to the entire structure and thus influencing the load path within this peculiar shell structure. In particular, we considered the interambulacral plate and species-specific skeletal pattern of *P. lividus* and selected different stereom types, including galleried stereoms at tubercle and suture, labyrinthic stereom at the plate centre and the perforate stereom at the basal zone (figure 1). A combination of micro-CT as well as geometric and elastic analyses was used to examine the elastic behaviour of these lightweight and strong microarchitectures.

## 2. Material and methods

Specimens of *P. lividus* were collected by scuba divers of the Stazione Zoologica Anton Dohrn in the marine protected area of Porto Cesareo, Italy ($40^{\circ }\;16.761^{\prime }$ N, $17^{\circ }\;51.292^{\prime }$ E; February 2023). Test samples were completely digested via treatment in 1 M NaOH (for 1 week) which completely removed the organic components and provided clean disassembled plates. The samples were then washed three times in deionized water to remove any loose organic debris as well as residual NaOH, and subsequently air-dried and successively analysed using X-ray microtomography.

### 2.1. X-ray microtomography

Microtomographic analyses were carried out using a Carl Zeiss Xradia Versa-410 3D X-ray microscope at the Istituto Nazionale di Geofisica e Vulcanologia, equipped with a polychromatic microfocus X-ray source (40–150kV maximum 10W), which allows the scanning of samples with a wide range of densities. This system includes a 2k×2k pixel, noise-suppressed charge-coupled detector equipped with different magnification objectives (0.4×, 4×, 10×, 20×), enabling high resolution, up to 0.9 μm per voxel. In this study, intact plates of $∼0.5\;{\text{cm}}^{2}$ from the treated echinoid tests were selected and scanned in absorption mode, acquiring 4001 projections over a $360^{\circ }$ rotation at 50–80kV and 4−7W, using 4× and 10× objectives, respectively. The resulting voxel size of the micro-CT images in this study ranges from ∼5.686 µm (4× objective) to ∼2.053 µm (10× objective). Scanning details are summarized in electronic supplementary material, table S2.

### 2.2. Data processing

**Figure 2.**
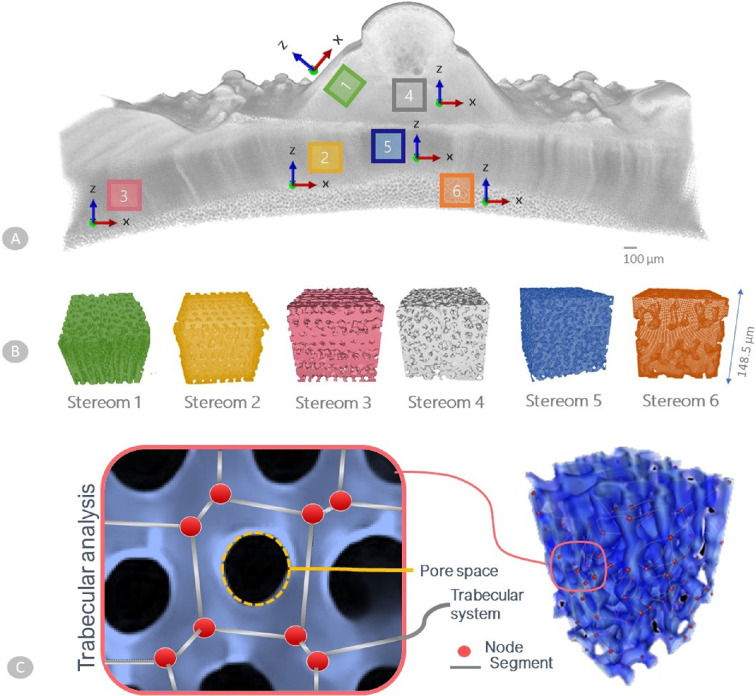
Stereom selection and trabecular analysis. (A) Micro-CT scan of the skeletal plate, showing the selected stereom sub-volume along with its corresponding *x, y, z* orientation. (B) 3D reconstructions of the selected stereom regions. (C) Trabecular analysis, highlighting the identification of pore spaces and the trabecular network, characterized by nodes and segments.

Micro-CT data were rendered and processed using ImageJ [48] and Seg3D software. Four sub-volumes were oriented, extracted and processed for six different plate regions corresponding to six different stereom types, namely: stereom 1, corresponding to galleried stereom located at tubercle boss; stereom 2, galleried stereom at suture area closer to the tubercle; stereom 3, galleried stereom at suture area closer to the plate’s edges; stereom 4, labyrinthic stereom located at plate centre; stereom 5, extracted from the plate centre and herein rediscovered as an angled galleried stereom; stereom 6, perforated stereom at the basal zone (figure 2A). Stereoms 2 and 3 have been considered to explore potential transitional effects on stereom geometry and stiffness along the plate. The imperforate stereom present at the mamelon zone has not been considered in this microstructural analysis. The 3D data were analysed using open-source Python libraries (i.e. SimpleITK, scikit-image, scipy, skan, openCV, numpy and pandas). Sub-volumes of interest (cubes of 100×100 pixels, corresponding to cubes of 148.5×148.5 µm) were extracted from the plate scan (electronic supplementary material, figure S1). The orientation used for extraction varies between stereom samples (specifically for stereom 1) and was determined based on the alignment of the galleries and the plate system to ensure consistency in the analysis, as shown in figure 2A. The size of these cubes was chosen to maximize the number of trabeculae analysed while avoiding transitional zones, ensuring focus on distinct stereomic regions and providing sufficient statistical representation (about 5000 trabeculae on average). Subsequently, median and Gaussian blur denoising filters were applied to the sub-volumes enhancing the contrast between material (stereom) and the surrounding non-material matrix. Sub-volumes were binarized via Otsu thresholding within Seg3D.

### 2.3. Trabecular geometric analysis

The binarized 3D sub-volume was skeletonized using an implementation of Lee *et al.* [49] from the scikit-image library. The skeletonization function transforms the trabecular system into a segment-node configuration: segments are lines equidistant to the boundaries of each trabecula describing their shapes, and nodes are the junctions or intersection points of these segments describing their connectivity. Node-segment configurations of the different microstructures were analysed to obtain key information regarding the trabecular system (table 1).

**Table 1.** Trabecular descriptors.

nodes (N)	number of connective points and related Euclidean coordinates (x,y,z)
segments (S)	number of trabeculae and related Euclidean coordinates of their constitutive points (x,y,z)
connectivity (C)	number of trabeculae connected to each node
chord length ($L_{c}$)	length of the straight line between two nodes
curved length ($L_{t}$)	length of the approximated curved course of a segment
tortuosity (τ)	ratio between curved length and chord length $\tau =L_{t}/L_{c}$
local thickness (T)	mean value of the minimum distance between the segment point and nearest pore calculated for each point describing a segment
porosity (P)	percentage of pore volume in the skeletal sub-volume compared with the entire sub-volume investigated

**Table 2.** Nomenclature used for FEM results.

E,ν	Young's modulus and Poisson's ratio of the basic organic calcitic material
$\epsilon ,\epsilon_{i}$	strain tensors and relevant components in Voigt notation
$\text{T},\sigma_{i}$	stress tensors and relevant components in Voigt notation
$\text{C},C_{ji}$	matrix representation and relevant components associated to the stiffness tensor, expressed in Voigt notation ($\sigma_{j}=C_{ji}\epsilon_{i}$)
${\text{C}}^{p},C_{ji}^{p}$	matrix representation and relevant components associated to the stiffness tensor in the principal reference, expressed in Voigt notation
${\text{n}}_{i}$	base unit vector of the stiffness principal reference. ${\text{n}}_{1}$ : direction of maximum longitudinal stiffness, ${\text{n}}_{3}$ : direction of minimum longitudinal stiffness, ${\text{n}}_{2}={\text{n}}_{3}\times {\text{n}}_{1}$ : direction of intermediate longitudinal stiffness
$\bar{□}$	average value of the quantity □ computed for all RVEs of a specific stereom type

### 2.4. Fabric anisotropy analysis

The material anisotropy can be represented by a symmetric, positive-definite, second-order tensor, denoted as fabric tensor, that describes the structural organization of material within a porous medium [50]. It can be estimated by a series of different approaches that represent a geometric analysis of the micro-CT data. In this study the fabric anisotropy of the different stereom types are quantified by the mean intercept length (MIL) method [51–54]. This technique evaluates the anisotropy and spatial organization of the calcite trabeculae within the stereom. Specifically, MIL calculates the average distance between material intersections (calcite and pores) along virtual linear paths sampled in various orientations, enabling a detailed assessment of structural anisotropy and porosity.

The quantitative analysis was conducted using Quant3D [53], specialized software for three-dimensional image processing and structural analysis. Quant3D streamlines the computation of MIL by generating a virtual grid of test lines within the micro-CT dataset and recording the frequency of intersections between the calcite matrix and the surrounding void spaces. This approach provided comprehensive insights into the stereom’s microarchitecture by computing the corresponding fabric tensor A for each analysed RVE. This tensor has components given in the local reference frame employed for each stereom type, as reported in figure 2A. The corresponding eigenvalues (i.e. the principal values of the fabric tensor $A_{11}$, $A_{22}$ and $A_{33}$) and eigenvectors (i.e. the principal directions ${\text{v}}_{1}$, ${\text{v}}_{2}$ and ${\text{v}}_{3}$ associated with the eigenvalues) were then evaluated to gain additional information about the material symmetries and orientations.

### 2.5. Identification of homogenized stiffness tensor components

The representation of material anisotropy by the fabric tensor alone is insufficient to fully characterize the elastic properties of micro-structured materials since this characterization provides a mechanical description applicable only to materials with three orthogonal planes of symmetry [50]. Moreover, the mechanical behaviour of stereoms under applied stresses is influenced by the elastic properties of the base material, which can exhibit inhomogeneity due to compositional and crystallographic variations in the Mg-calcite. Thus, to evaluate the response of each architectural type to external forces and deformations, stereoms can be analysed by neglecting the unknown calcite variability and assuming a homogeneous calcitic material. The elastic properties of the base material were assumed to be linearly elastic, with a Young’s modulus of E=73500MPa and a Poisson’s ratio ν=0.4, corresponding to solid organic calcite [55].

Linear elastic FEA was conducted to evaluate the mechanical response of a set of representative volume elements (RVE) derived from the four selected sub-volumes of each stereom type. The homogenized stiffness tensor components for each model were computed by averaging the stress–strain relationship within each corresponding RVE. In particular, the geometries of the RVEs were obtained as isosurfaces from the binarized 3D sub-volumes extracted with Seg3D and then processed via SIMULIA Abaqus software. These isosurfaces were meshed using tetrahedral/hexahedral finite elements. To minimize the approximations introduced by finite element discretization, a mesh convergence analysis was conducted for each RVE to determine an appropriate element size. On average, each model comprises approximately 5 million nodes and 3 million second-order (quadratic) tetrahedral elements. Mesh quality was validated using the collapse test, ensuring that the tetrahedral collapse index remained above 0.20 in all models. To reduce computational time, the analyses were performed using parallel computing on an Intel Core i7 CPU, with 4 cores dedicated to the process. The computational time for each model was approximately 5 min.

**Figure 3.**
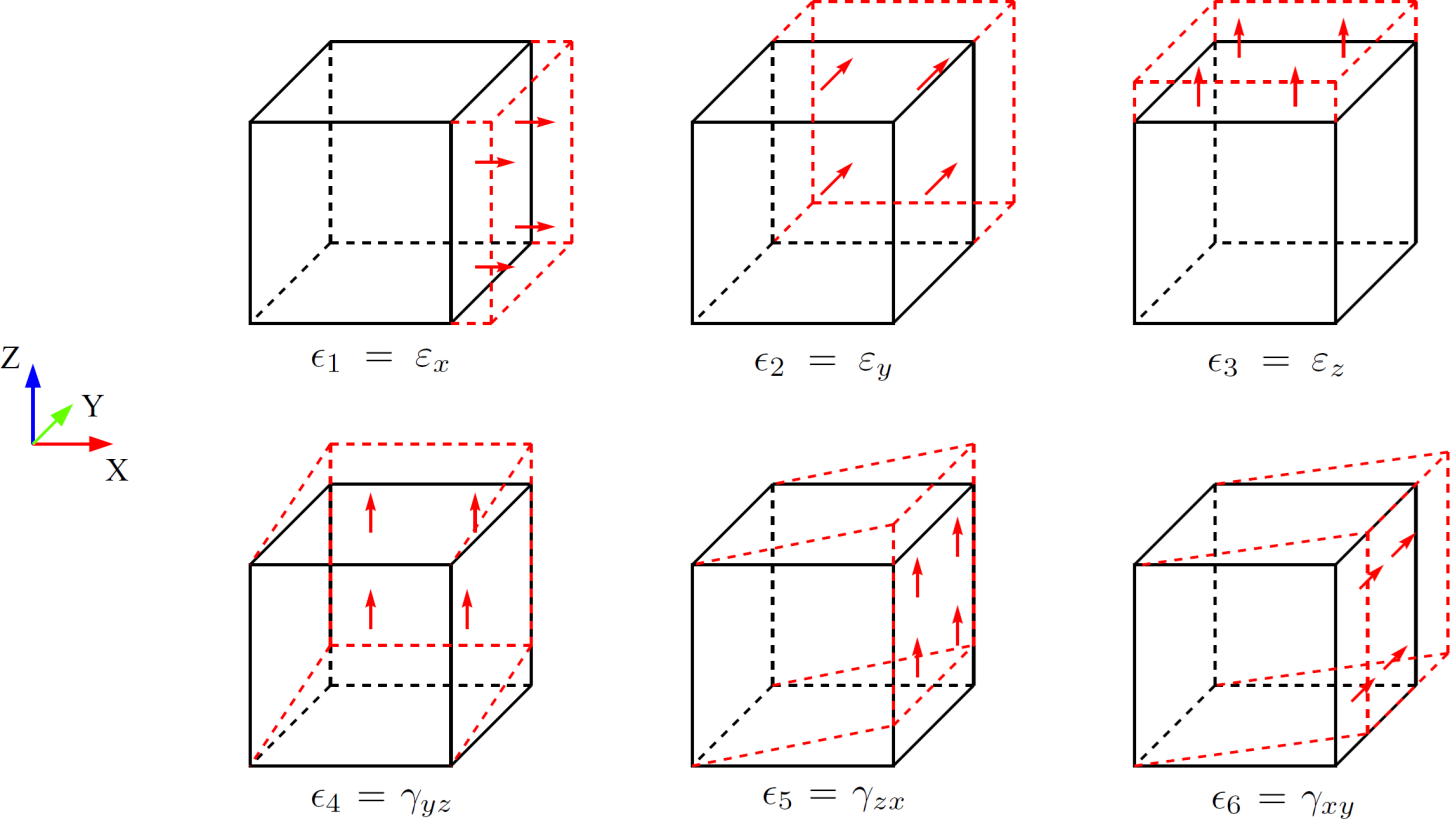
Boundary conditions for tensile and shear loading in FEM.

Once the finite element model of RVEs was set, six analyses were performed by applying a uniform distribution of a single strain component $\epsilon_{i}=0.00211$, i=1,2,...,6, while keeping the other strain components equal to zero. This analysis used the Voigt notation, in which $\epsilon_{1}=\varepsilon_{x}$, $\epsilon_{2}=\varepsilon_{y}$ and $\epsilon_{3}=\varepsilon_{z}$ represent linear strains, while $\epsilon_{4}=\gamma_{yz}$, $\epsilon_{5}=\gamma_{zx}$ and $\epsilon_{6}=\gamma_{xy}$ indicate shear strains. Figure 3 illustrates the set of Dirichlet kinematic boundary conditions applied to each case. These boundary conditions were applied separately to each RVE, and the average stress and strain were computed. While this procedure tends to overestimate the microstructure stiffness, the approximation becomes negligible for bone volume fractions above 30% [56]. Since bone volume fraction is estimated as the complement of porosity, it remains above 40% for all RVEs analysed.

The results of the generic *i*-th analysis were used to compute the components of the homogenized stiffness tensor associated with the corresponding stereom as


(2.1) $$\begin{array}{lcr} & C_{ji}=\frac{{\bar{\sigma }}_{j}}{\epsilon_{i}}\;\text{where}\;{\bar{\sigma }}_{j}=\frac{\int_{V}\sigma_{j}\;dV}{\int_{V}dV},\;j=1,2,...,6 & \end{array}$$

${\bar{\sigma }}_{j}$ being the average value of the stress component $\sigma_{j}$ computed on the entire volume V of the stereom RVE. The resulting homogenized stiffness tensors were expected to exhibit anisotropic components. It is possible to identify the directions of higher and lower longitudinal stiffness, represented by the directions ${\text{n}}_{1}$ and ${\text{n}}_{3}$ along which $C_{11}$ is maximized or minimized, respectively.

To determine the maximum and minimum values of $C_{11}$ and the corresponding directions, a set of three orthogonal unit vectors was generated, spanning the range of polar and azimuthal angles with intervals of 1 degree. The components of C are then computed in the rotated reference frame defined by these unit vectors as basis. To this end, the stiffness tensor C is expressed in the rotated reference using the rotation formula ${\text{C}}^{\prime }=\text{M}\text{C}{\text{M}}^{T}$, where [57]:


(2.2) $$\text{M}=\left[\begin{array}{cccccc} i_{x}^{2} & j_{x}^{2} & k_{x}^{2} & 2j_{x}k_{x} & 2i_{x}k_{x} & 2i_{x}j_{x}\\ i_{y}^{2} & j_{y}^{2} & k_{y}^{2} & 2j_{y}k_{y} & 2i_{y}k_{y} & 2i_{y}j_{y}\\ i_{z}^{2} & j_{z}^{2} & k_{z}^{2} & 2j_{z}k_{z} & 2i_{z}k_{z} & 2i_{z}j_{z}\\ i_{y}i_{z} & j_{y}j_{z} & k_{y}k_{z} & j_{y}k_{z}+k_{y}j_{z} & i_{y}k_{z}+k_{y}i_{z} & i_{y}j_{z}+j_{y}i_{z}\\ i_{x}i_{z} & j_{x}j_{z} & k_{x}k_{z} & j_{x}k_{z}+k_{x}j_{z} & i_{x}k_{z}+k_{x}i_{z} & i_{x}j_{z}+j_{x}i_{z}\\ i_{x}i_{y} & j_{x}j_{y} & k_{x}k_{y} & j_{x}k_{y}+k_{x}j_{y} & i_{x}k_{y}+k_{x}i_{y} & i_{x}j_{y}+j_{x}i_{y} \end{array}\right]$$

while $\text{i}=(i_{x},\;i_{y},\;i_{z})$, $\text{j}=(j_{x},\;j_{y},\;j_{z})$ and $\text{k}=(k_{x},\;k_{y},\;k_{z})$ are the unit vectors representing the ortho-normal base of the new reference. Accordingly, ${\text{n}}_{1}$ and ${\text{n}}_{3}$ are set equal to the unit vectors i that produce, respectively, the minimum and maximum values of $C_{11}^{\prime }$.

Once the unit vectors ${\text{n}}_{1}$ and ${\text{n}}_{3}$ were determined, the third axis of the principal reference for the homogenized material, namely ${\text{n}}_{2}$, was defined as the one that minimizes the determinant of the minor of C representing the longitudinal-shear coupling. Such procedure avoids any preliminary hypothesis about the material symmetry. Actually, material orthotropy can be verified by checking that $n_{1}$, $n_{2}$, $n_{3}$ are mutually orthogonal and evaluating any coupling between longitudinal and shear behaviour.

Successively, the components of C in the principal material reference frame, denoted by $C_{ij}^{p}$, were calculated using the rotation formula with $\text{i}={\text{n}}_{1}$, $\text{j}={\text{n}}_{2}$ and $\text{k}={\text{n}}_{3}$$\text{k}={\text{n}}_{3}$. To obtain representative values for the directions of the principal material reference and the principal stiffness tensor components, their average values were computed for each stereom type to account for intra-typological material inhomogeneity.

Engineering material parameters are also computed as a function of the Compliant tensor ${\text{S}}^{p}=({\tilde{\text{C}}}^{p}{)}^{-1}$, ${\tilde{\text{C}}}^{p}$ being the stiffness tensor expressed in Kelvin’s notation, see, e.g. [58]. Accordingly, the Young’s moduli are computed as $E_{ii}=1/S_{ii}^{p}$, i=1,...,3, while the shear moduli are determined as $G_{23}=C_{44}^{p}$, $G_{31}=C_{55}^{p}$ and $G_{12}=C_{66}^{p}$. The Poisson’s ratios $\nu_{kl}$, which represent the coupling between the strain along the loaded direction $\epsilon_{l}$ and the one produced along a transversal direction $\epsilon_{k}$, are evaluated as:


(2.3) $$\begin{array}{lcr} & \left[\begin{array}{ccc} -1 & \nu_{12} & \nu_{13}\\ \nu_{21} & -1 & \nu_{23}\\ \nu_{31} & \nu_{32} & -1 \end{array}\right]=-\left[\begin{array}{ccc} E_{11} & 0 & 0\\ 0 & E_{22} & 0\\ 0 & 0 & E_{33} \end{array}\right]\left[\begin{array}{ccc} S_{11}^{p} & S_{12}^{p} & S_{13}^{p}\\ S_{21}^{p} & S_{22}^{p} & S_{23}^{p}\\ S_{31}^{p} & S_{32}^{p} & S_{33}^{p} \end{array}\right] & \end{array}$$

To further highlight the elastic behaviour of the analysed stereoms, a 3D representation of the relationship between each elastic tensor component and the orientation of the reference frame was generated. From the shape of these 3D directional representations, it is possible to visualize the anisotropic behaviour of the material [59,60]. In particular, the 3D representation of longitudinal stiffness components in an isotropic material appears as a spherical surface, while an elongated surface indicates a higher level of elastic anisotropy.

### 2.6. Analysis of the relationship between fabric and stiffness tensors

The relationship between the eigenvalues of the fabric tensor A and the components of the elastic stiffness tensor of the material C was established by Cowin in [50]. Such relationship is valid for orthotropic, transversely isotropic or isotropic materials only. Hence any employment of the fabric tensor to characterize the material anisotropy requires a preliminary hypothesis about the material symmetry.

In Cowin’s original formulation, the relationship between A and C is defined by nine parameters $a_{1}$,..., $c_{3}$. These parameters offer a more comprehensive characterization of the material, as the fabric tensor alone describes only the geometric arrangement of the material, not its actual stiffness.

If both the fabric tensor and the elastic stiffness matrix are available, and provided that C exhibits at least three orthogonal planes of symmetry, the nine parameters $a_{1}$, ..., $c_{3}$ can be determined by solving a system of nine linear equations, where the coefficients are nonlinear functions of the fabric tensor’s eigenvalues. However, the coefficient matrix of this system is numerically singular, leading to an imprecise solution. Additionally, the relationship varies depending on whether the material is orthotropic, transversely isotropic or isotropic, without a smooth transition between these behaviours.

To address these limitations, a new relationship between the fabric tensor and the elastic stiffness matrix was proposed by Moesen *et al*. in [58], introducing nine new parameters $p_{1}$, ..., $p_{9}$. Determining these parameters requires the evaluation of the deviatoric component of the fabric tensor and the definition of an orthogonal reference frame in which C is expressed. In our study, we employed the MMTensor MATLAB code provided in [61] to compute these parameters following their proposed procedure.

The definition of the orthogonal basis employed in such analysis has variable arbitrarity, depending on the material symmetry. Moesen *et al*. divided the basis vectors into three groups corresponding to decreasing material symmetry. In particular, the first two vectors are selected for isotropic materials, additional three vectors are indicated for the transversely isotropic case and further four vectors are added for orthotropic materials.

### 2.7. Statistical analysis

To investigate the specific differences between various stereom structures, non-parametric Kruskal–Wallis tests were performed on trabecular data, MIL fabric eigenvalues, principal longitudinal stiffness components and engineering constants, using PAST software for Windows (version 4.16). Specific statistical differences between the identified galleried stereoms (stereoms 1 and 2, stereoms 2 and 3, stereoms 2 and 5) were assessed using the two-sample Wilcoxon Mann–Whitney test. These pairwise comparisons were selected because stereom types share a galleried microstructure and similar visual features, making statistical testing essential to discern subtle but meaningful differences pairwise comparisons were selected because stereom types share a galleried microstructure and similar visual features, making statistical testing essential to discern subtle but meaningful differences. To visualize the distinctions among stereom types, non-metric multidimensional scaling (nMDS) ordination was employed for trabecular, fabric and stiffness results. Differences and similarities between the stiffness components were verified by comparing mean values and standard deviations of each group of results for each stereom types. The corresponding results were successively visualized in the form of box plots representation of the distribution of stiffness components values plots representation of the distribution of stiffness components values. Finally, correlation analyses were conducted to evaluate the strength of linear relationships between the trabecular variables, the principal longitudinal stiffness components and engineering constants using Pearson’s r linear correlation statistics.

## 3. Results

### 3.1. Trabecular analysis

**Figure 4.**
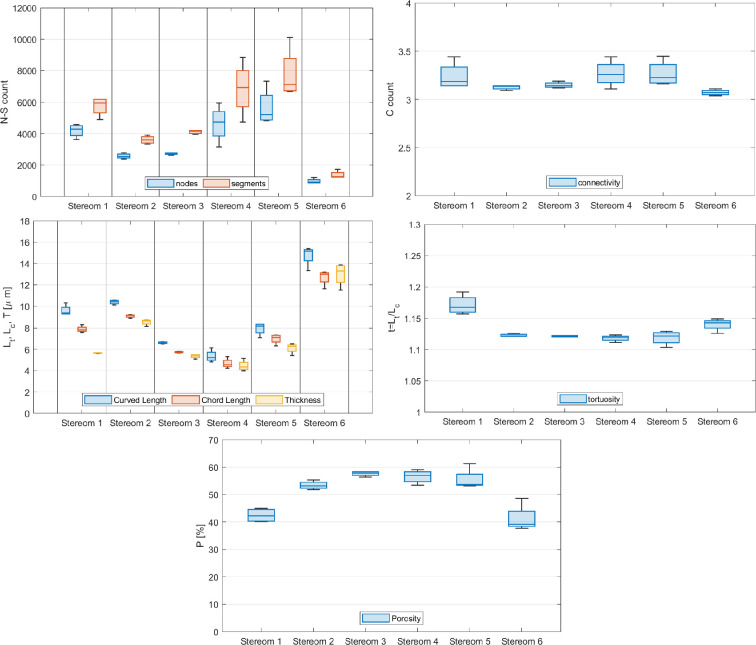
Trabecular analysis. Box plots of node–segment configuration, descriptors of the trabecular system, tortuosity, connectivity and porosity comparing the investigated stereom types.

The results of the segment-node configuration and the trabecular analysis are summarized in figure 4 and electronic supplementary material, table S3, comparing the different stereom types. All stereoms show specific length of trabeculae and related thickness with a little tortuosity and similar values of connectivity of about 3. Among them, a clear divergence can be detected in stereom 6, characterized by a smaller number of nodes and segment and larger (longer and thicker) trabeculae with respect to the other stereom types. The porosity is lower in stereoms 1 and 6, while it is higher for stereoms 2–5, corresponding to the central part of the plate. The Kruskal–Wallis tests identified statistically significant variations among the stereom types analysed, with a significance threshold of α=5%. In the trabecular analysis, parameters such as nodes, points, segments, curved length, chord length, tortuosity, thickness and porosity exhibited significant differences across the stereom types (electronic supplementary material, table S4). When comparing stereom types 1 and 2 using the two-sample Wilcoxon Mann–Whitney test, significant differences were observed in nodes, segments, curved length, chord length, tortuosity, thickness and porosity (*p* = 0.03). However, connectivity and curved length were not statistically significant (electronic supplementary material, table S5). Comparisons between stereom types 2 and 3 revealed that segments, curved length, chord length, thickness, porosity circularity differed significantly (p=0.03), whereas nodes, tortuosity and connectivity did not show a statistically significant difference (electronic supplementary material, table S5). Comparisons between stereom types 2 and 5 showed that nodes, segments, curved length, chord length, thickness and connectivity are significant different (p≤0.03), while tortuosity and porosity did not show any differences. The spatial differences between the stereom types for the skeleton data were also evidenced by the nMDS plot (electronic supplementary material, figure S2), which showed a clear separation between the six types. Stereom 3 is really close to the labyrinthic stereom as reported in the 2D analysis [47]. This has been reported in the 2D analysis as also the interrelationships among geometrical features, such as curved length and chord length showed a nearly perfect positive correlation, indicating proportional scaling of these parameters. Thickness was positively correlated with both curved length and chord length suggesting a strong interdependence (electronic supplementary material, table S6).

### 3.2. Fabric anisotropy

**Figure 5.**
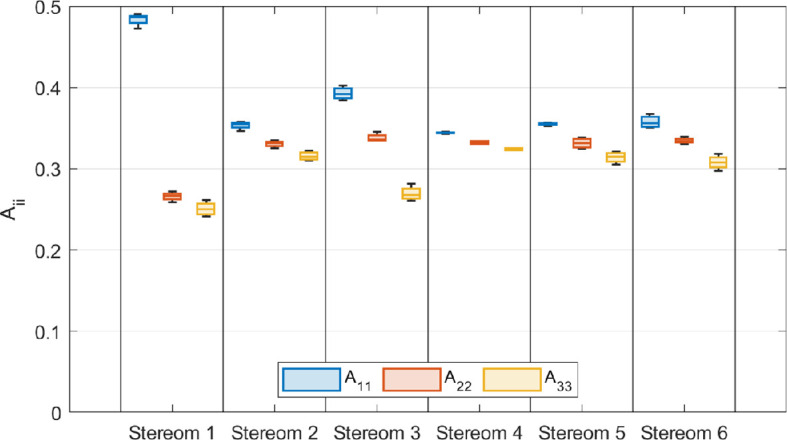
Averaged eigenvalues of fabric tensor eigenvalues for each stereom type.

The fabric analysis results are summarized in figure 5, which presents the average fabric eigenvalues for each stereom type. Detailed numerical values of the fabric tensor eigenvalues and eigenvectors for all RVEs across all stereom types are provided in the supplementary material (electronic supplementary material, tables S7–S19).

These results indicate that stereom 1 is approximately transversely isotropic, with $A_{11}$ significantly larger than $A_{22}$ and $A_{33}$, which are nearly equal. The eigenvector ${\text{v}}_{1}$, corresponding to the largest eigenvalue $A_{11}$, aligns with the local z-axis.

By contrast, stereom 3 exhibits pronounced orthotropy, with three distinct fabric tensor eigenvalues. The eigenvector ${\text{v}}_{1}$ is parallel to the local x-axis, while ${\text{v}}_{2}$ and ${\text{v}}_{3}$ lie within the y−z plane, with $v_{2}$ positioned closer to the local z-axis.

Stereoms 2, 5 and 6 exhibit a comparable degree of orthotropy, with distinct fabric tensor eigenvalues, though their differences are less pronounced than in stereom 3. In stereoms 2 and 6, $v_{1}$ aligns with the local z-axis, whereas in stereom 5, ${\text{v}}_{1}$ is inclined and varies between different RVEs.

Finally, stereom 4 displays the lowest anisotropy, with nearly equal fabric tensor eigenvalues, indicating an approximately isotropic microstructure. Across all RVEs of stereom 4, ${\text{v}}_{1}$ remains closer to the local z-axis. The Kruskal–Wallis tests revealed significant statistical differences in eigenvalues across all analysed stereom types (*p* = 0.001) (electronic supplementary material, table S20). Pairwise two-sample Wilcoxon Mann–Whitney tests showed significant differences between stereoms 1 and 2 (*p* = 0.03) and between stereoms 2 and 3 (*p* = 0.03), but no significant differences were found between stereoms 2 and 5 (*p* = 0.88) (electronic supplementary material, table S21). nMDS plots indicated that stereoms 1 and 3 exhibited the greatest divergence compared with all other stereom types (electronic supplementary material, table S2). The correlation analysis revealed significant interconnections between the fabric tensor eigenvalues, tortuosity and porosity (electronic supplementary material, tables S22−S23).

### 3.3. Homogenized stiffness tensor components and engineering parameters

The averaged stiffness tensor components, expressed in the reference frame of maximum–minimum longitudinal stiffness, indicate that all stereom types exhibit at least orthotropic material symmetry. This is evidenced by the fact that both normal-shear and shear–shear coupling values are approximately zero (below 0.5% of $C_{11}^{p}$), as detailed in the electronic supplementary material, tables S24−S35.

**Figure 6.**
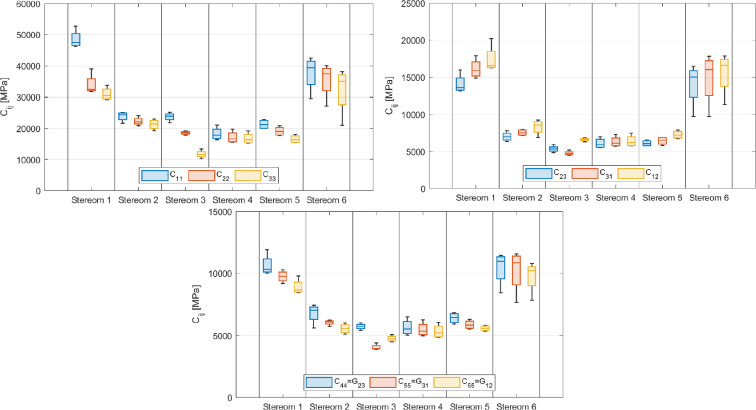
Principal longitudinal stiffness components (top left), principal longitudinal stiffness coupling components (top right) and principal shear stiffness components (bottom) for each stereom type.

The nonzero stiffness components are illustrated in figure 6 using three box plots, which respectively display the average principal values of longitudinal stiffness, longitudinal coupling and shear stiffness for all stereom types.

**Figure 7.**
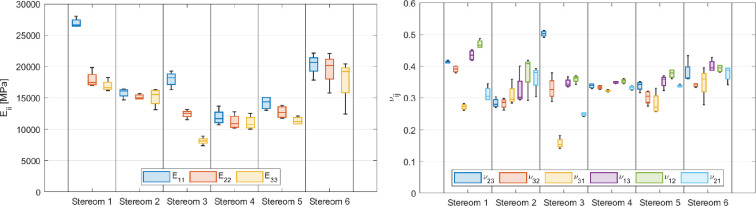
Averaged values of engineering material parameters for each stereom type: Young's moduli (left) and Poisson's ratios (right).

Additionally, the corresponding average values of engineering material parameters, including Young’s moduli ($E_{ii}$), shear moduli ($G_{ij}$) and Poisson’s ratios ($\nu_{ij}$), are summarized in figures 6 and 7. The complete dataset, including values for all RVEs of each stereom type, is provided in tabular format in the electronic supplementary material, tables S36–S41).

Varying degrees of orthotropy are evident across different stereom types, each exhibiting distinct stiffness characteristics. Stereoms 1 and 6 have remarkably higher stiffness, clearly indicated by the higher values of their stiffness components and Young’s moduli. Stereoms 1 and 3 demonstrate a pronounced stiffness specialization, with a clear anisotropic behaviour. While the values of longitudinal stiffness and Young’s moduli of stereom 1 manifest its almost transversely isotropic behaviour ($C_{11}^{p}>C_{22}^{p}\approx C_{33}^{p}$ and $E_{11}>E_{22}\approx E_{33}$), stereom 3 is markedly orthotropic ($C_{11}^{p}>C_{22}^{p}>C_{33}^{p}$ and $E_{11}>E_{22}>E_{33}$). When analysing their longitudinal coupling and shear stiffness, instead, stereom 1 manifests orthotropy ($C_{12}^{p}>C_{13}^{p}>C_{23}^{p}$ and $C_{44}^{p}=G_{23}>C_{55}^{p}=G_{31}>C_{66}^{p}=G_{12}$), while stereom 3 shows an approximately transversely isotropic material symmetry ($C_{12}^{p}>C_{13}^{p}\approx C_{23}^{p}$ and $C_{44}^{p}=G_{23}>C_{55}^{p}=G_{31}\approx C_{66}^{p}=G_{12}$). Poisson’s ratios of these stereom types exhibit large variability along the six direction couples, and in both cases $\nu_{31}$ and $\nu_{21}$ are lower than the others.

The stiffness components of stereoms 2, 5 and 6 manifest similar differences along the principal material directions. The differences between these three stereom types are more pronounced when observed in terms of engineering parameters. In particular, among these three microstructures, stereom 5 exhibits the largest differences between the Young’s moduli along the three material directions while stereom 2 exhibits the largest differences between the Poisson’s ratios along the six direction couples. Stereom 4, instead, appears nearly isotropic, as both the stiffness components and engineering parameters are relatively similar in magnitude along all material directions.

The Kruskal–Wallis tests identified statistically significant variations among the stereom types analysed for the principal longitudinal stiffness components ${\bar{C}}_{11}^{p}$, ${\bar{C}}_{22}^{p}$, ${\bar{C}}_{33}^{p}$, ${\bar{C}}_{12}^{p}$, ${\bar{C}}_{13}^{p}$, ${\bar{C}}_{23}^{p}$, ${\bar{C}}_{44}^{p}$, ${\bar{C}}_{55}^{p}$ and ${\bar{C}}_{66}^{p}$ (electronic supplementary material, table S43). Two-sample Wilcoxon Mann–Whitney test comparing stereom types 1 and 2 shows significant differences in all stiffness components (*p* = 0.03) (electronic supplementary material, table S44). Conversely, the comparison between stereom types 2 and 3 revealed that all the stiffness components differed significantly, whereas ${\bar{C}}_{11}^{p}$ and ${\bar{C}}_{44}^{p}$ did not show a statistically significant difference (electronic supplementary material, table S44). Interestingly, comparisons between stereom types 2 and 5 did not show a statistically significant difference, besides ${\bar{C}}_{33}^{p}$ and ${\bar{C}}_{13}^{p}$ (electronic supplementary material, table S44). The spatial variations in stiffness values among the different stereom types were also highlighted by the nMDS plot (electronic supplementary material, figure S2), which revealed a clear divergence of stereom types 1, 3 and 6, while stereom types 2, 4 and 5 exhibited greater similarity.

The correlation analysis revealed statistically significant relationships between microstructural parameters and stiffness components (electronic supplementary material, tables S45−S46). Among all variables, porosity showed a strong negative correlation with stiffness values. The curved length, chord length, tortuosity and thickness also exhibited positive correlations with stiffness parameters (electronic supplementary material, tables S45−S46).

### 3.4. Relationship between fabric and stiffness tensors

**Figure 8.**
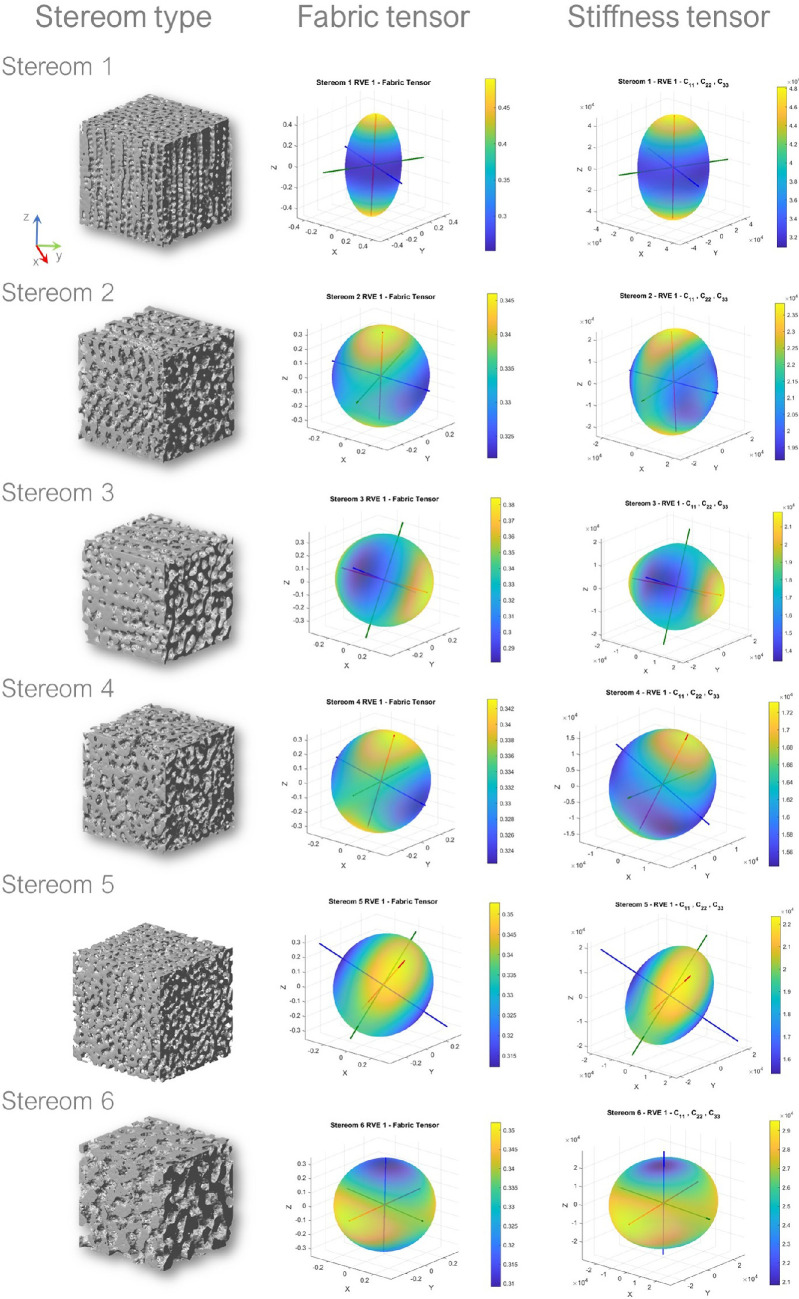
Micro-CT scans of the analysed stereom types (RVE1) (left column) and related 3D directional representation of fabric tensor (centre column) and longitudinal components of the stiffness tensor (right column).

Material symmetries deducible from the fabric analysis and from the stiffness characterization are qualitatively similar for all stereom types figure 8. In particular, both analyses show that stereom 1 is approximately transversely isotropic, with ${\text{v}}_{1}\approx {\text{n}}_{1}$ directed along the local z-axis, while stereom 3 is markedly orthotropic with ${\text{v}}_{1}\approx {\text{n}}_{1}$ directed along the local x-axis. According to both analyses, stereoms 2, 5 and 6 also exhibit orthotropic symmetry, though characterized by a lower degree of anisotropy. The material directions computed from the fabric analysis are similar to those computed from the stiffness characterization for all these stereom types. Finally, both analyses show that stereom 4 is approximately isotropic. Although less relevant in this case, the two analyses produce similar sets of principal material directions for stereom 4.

The results of the fabric analysis and the stiffness characterization can be qualitatively compared from figure 8. Here the RVE 1 of all stereom types is shown by the 3D view of its microstructure, the corresponding ellipsoidal representation of the fabric tensor and the directional representation of the longitudinal stiffness components. The elongated shape of the 3D surfaces associated with stereoms 1 and 3 confirms their high specialization towards the axis of maximum longitudinal stiffness. By contrast, stereom 4, which is almost isotropic, features a nearly spherical surface. The red, green and blue axes in the figure indicate the orientation of the principal material directions related with the local x,y,z.

The fabric tensor provides only a partial representation of the complex elastic behaviour of each stereom type. For example, the higher stiffness of stereoms 1 and 6, as well as the intricate shear and longitudinal coupling behaviours of stereoms 1 and 3, can only be fully understood through a comprehensive stiffness characterization of the microstructure.

Although Cowin proposed a relationship between the fabric and stiffness tensors [50], computing the stiffness tensor as a function of the fabric tensor requires knowledge of nine material parameters $a_{1}$, ..., $c_{3}$, which depend on both the intrinsic properties of the base material and the porosity of the microstructure. In our analysis, we estimated these parameters based on the computed fabric and stiffness tensors for each RVE. This allowed us to quantitatively compare the two sets of results by determining the material parameters that govern the relationship between the fabric and stiffness tensors.

The evaluation of Cowin’s parameters exhibits significant instability, as previously noted in [58] (electronic supplementary material, tables S52−S64). In our analysis, these parameters showed high dispersion across the RVEs of each stereom type. By contrast, the alternative parameters $p_{1}$, ..., $p_{9}$ introduced in [58] demonstrated lower numerical instability, with more consistent values, particularly in stereom types with a higher degree of anisotropy. However, the variation of these parameters progressively increases from $p_{1}$ to $p_{9}$. This numerical instability arises from the challenge of selecting an appropriately ordered basis that minimizes the polynomial degree required to compute the eigenvalues of the fabric-stiffness coefficient matrix. Unavoidable survey and numerical approximations in computing both the fabric and stiffness tensors further contribute to this issue.

The correlation analysis revealed a strong correlation between the $p_{1}$, $p_{2}$ and porosity, as well as with curved length, chord length and tortuosity. The significance of the correlation decreases from $p_{1}$ to $p_{9}$, probably due to the increasing variability and instability identified in the parameter (electronic supplementary material, tables S67–S68).

## 4. Discussion

In this study, the microstructural and regional stiffness variation of the inter-ambulacral plates of *P. lividus* were determined in a 3D coordinate system, leading to new and interesting insights. Specifically, X-ray microtomography was used to retrieve the 3D geometric features of a series of representative volumes of the stereom extracted from different regions of the inter-ambulacral plates of *P. lividus*. These scans were analysed to obtain geometric and elastic information regarding the trabecular system that characterizes each analysed stereom type. Each stereom showed unique topological features and variable porosity between (41.15±5.0%) (stereom 6) and (56.55±2.4%) (stereom 4), similar to the values detected in the periodic stereom at the tubercle of the sea urchin *Phyllacanthus imperialis* (53.2%) [45] and radiating layer (38±3.8%) and the medulla of *Heterocentrotus mamillatus* (61±4.8%) [62]. Fabric and stiffness characterization confirmed and highlighted important differences among the different stereom types with respect to their trabecular configuration and elastic behaviour.

### 4.1. Stereom 1: the galleried stereom located at the tubercle boss

Stereom 1 corresponds to the galleried stereom located at the tubercle boss and is characterized by numerous trabeculae aligned with the direction orthogonal to the tubercle lateral surface. The stereom consists of numerous parallel pores visible in the *zx* and *zy* planes (figure 8A), which have been previously described to be arranged as a Voronoi construction with a prevalence of hexagonal polygons, and a regularly organized seed distribution [63]. Moreover, as previously reported in the 2D analysis [47], the 3D geometric analysis also confirms that this galleried stereom statistically differs from stereoms 2 and 5 in terms of number of nodes, segments, curved length, chord length, thickness, connectivity and porosity. The high values of $E_{11}$ ($C_{11}^{p}$, or $A_{11}$) with respect to $E_{22}$ and $E_{33}$ ($C_{22}^{p}$, $C_{33}^{p}$ or $A_{22}$, $A_{33}$) characterize all RVEs and highlight the anisotropic behaviour and the directional stiffness specialization of stereom 1. This is confirmed by the elongated shape of the 3D surfaces representing the directional variation of the longitudinal stiffness components (figure 8E). The Poisson’s effect is characterized by weaker coupling between stresses applied along ${\text{n}}_{1}$ and the resulting strains along $n_{2}$ and ${\text{n}}_{3}$, as indicated by the lower values of the Poisson’s ratios $\nu_{31}$ and $\nu_{21}$. Shear effects, on the other hand, are marked by greater stiffness in the ${\text{n}}_{2}-{\text{n}}_{3}$ plane, as reflected by the higher values of $G_{23}$.

The direction of the first fabric eigenvalue (${\text{v}}_{1}$) aligns with that of the maximum longitudinal stiffness (${\text{n}}_{1}$) as shown in figure 8. This direction is normal to the lateral surface of the tubercle. The two directions associated to the second and third fabric eigenvalue (${\text{v}}_{2}$ and ${\text{v}}_{2}$), which align with those corresponding to lower longitudinal stiffness (${\text{n}}_{2}$ and ${\text{n}}_{3}$) lie on the plane tangential to this surface (electronic supplementary material, figure S16). Such high uni-directional specialization in stereom 1, which can effectively transfer stresses along the direction ${\text{n}}_{1}$, presents clear insights into the function of the tubercle stereom in living organisms. Indeed, this stereom offers an attachment site for the catch apparatus fibres connecting the spine to the tubercle, which is therefore subjected to intense directional and impact stresses [25,45,64,65]. Recently [45], reported how the periodic stereom structures at the tubercle of the sea urchin *Phyllacanthus imperialis* provide an excellent fracture resistance and energy dissipation capacity. Another characteristic of stereom 1, which is not fully investigated and requires additional analysis, is related to its growth banding. This banding could presumably influence the mechanical response of the tubercle, as has already been assessed in echinoid spines. Studies have shown that variations in mineral density within growth bands can impact mechanical and structural properties [38,66,67]. In particular, research on echinoid spines has demonstrated that those exhibiting growth rings are capable of withstanding greater forces compared with spines without them [37,38,67].

### 4.2. Stereoms 2 and 3: the non-uniform galleried stereom located at the suture region

The microstructure identified by Smith [14] as galleried stereom located at the suture region exhibits a gradual variation of geometry. In our study, we selected two representative stereom types, namely stereoms 2 and 3, by extracting eight RVEs from this apparently uniform microstructure. Four of the RVEs, those associated with stereom 2, were extracted near the tubercle, while the others, associated with stereom 3, were taken closer to the plate sutures. All RVEs exhibited a microstructure characterized by pores that are parallelly organized in the *yx* and *zx* planes (figure 8). A visual analysis of the two sets of RVEs shows their similarity since both stereom types are characterized by galleries parallel to the x axis. However, differently from stereom 3, stereom 2 presents some elongated elements parallel to y and z (see, e.g. figure 8, centre and right columns).

Although they may show a similar number of nodes and connections, the trabecular length, thickness and porosity are significantly different in these stereoms (figure 4) (electronic supplementary material, table S5). Furthermore, their stiffness characterization indicates that these stereoms exhibit very different elastic behaviour, despite the visual similarity. In particular, the Young’s modulus $E_{11}$ ($C_{11}^{p}$ and $A_{11}$) of all RVEs is significantly higher than the corresponding moduli along the other principal material directions. Conversely, stereom 2 is characterized by a much less anisotropic behaviour (see, e.g. the electronic supplementary material). The orthotropic nature of stereom 3 is evident from the distinctly different shear stiffness values, which consistently satisfy the condition $G_{23}>G_{12}>G_{31}$ across all RVEs (figure 7). Additionally, its Poisson’s ratios exhibit a unique pattern: $\nu_{13}$, $\nu_{12}$ and $\nu_{32}$ are noticeably lower than $\nu_{23}$ but higher than $\nu_{31}$, and $\nu_{21}$ (figure 7). Stereom 2 is almost isotropic, although it exhibits a maximum longitudinal stiffness in the direction orthogonal to plate mid-surface (z), which is interestingly orthogonal to the direction of galleries (x) and aligned with the elongated elements visible in figure 8A,B. The stiffer direction (${\text{n}}_{1}$) of all RVEs of stereom 3, instead, is parallel to galleries (x) and coincident with the longitudinal direction of the plate. The direction associated with the intermediate principal value of the longitudinal stiffness, ${\text{n}}_{2}$, is orthogonal to the plate mid-surface (y). The compliant direction ($n_{3}$) is directed along the plate transversal direction (z).

**Figure 9.**
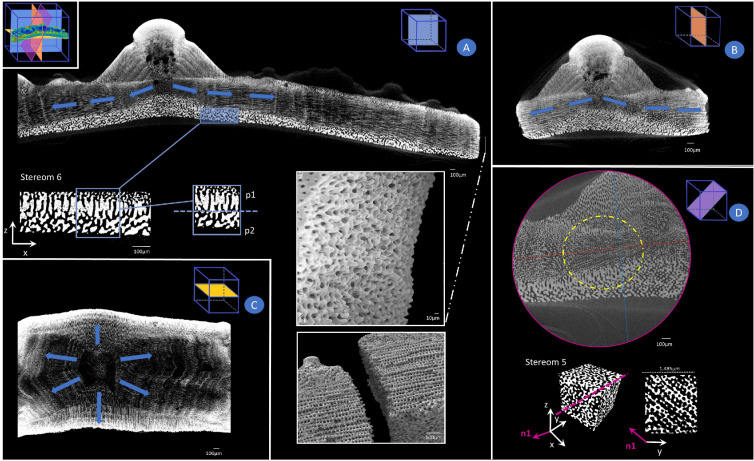
Micro-CT scan of *P. lividus* plate from different plane perspectives: (A) Coronal plane, highlighting the perforated stereom 6 with detailed views of its two sub-layers (p1 and p2), as well as the suture area featuring skeletal protrusions (finger joints). (B) transversal plane and (C) sagittal plane, showing the galleries departing from the centre of the primary tubercle with growth bands (indicated by blue arrows). (D) Angled plane relative to the coronal plane, revealing the galleried structure of stereom 5 and a representative RVE, with a layer cut at 63°, exposing the angled galleries.

The differences between stereom types 2 and 3 show that galleried stereom is characterized by an increased stiffness specialization while moving from the plate centre towards its edges. This may be associated with the necessity to directionally transfer the stress from the spine actions towards the suture. Actually, the coronal, sagittal and transversal sections of the entire plate shown in figure 9A–C display the varying orientation of the galleried stereom departing from the plate centre towards the plate edges. Studies in the literature have reported that the trabeculae with a periodic arrangement are able to disseminate applied stresses directionally onto the adjacent ones, decreasing the chance of a localized structural failure [63,68]. This suggests a significant adaptive advantage, particularly at the edges (stereom 3), where the presence of collagen fibres provides flexibility that might aid in stress dissipation, ultimately optimizing the macro-mechanical behaviour of the entire echinoid test [10].

### 4.3. Stereoms 4 and 5: the labyrinthic stereom located at the tubercle centre and the transition zone towards an inclined galleried microstructure

In its plate model [14], Smith reported a labyrinthic stereom at the centre of the tubercle and at the plate centre which seems to be classified as a unique microstructure, as in fact seems apparently deducible from a 2D perspective [47]. We extracted four RVEs from both these locations, namely stereom 4, at the centre of the tubercle, and stereom 5, located at the centre of the plate, just underneath the tubercle. Both stereoms present a geometry which is apparently not directional if observed on the principal anatomical planes of the inter-ambulacral plate (see, e.g. figure 8A–D). However, the different material scale visible in such views produces the geometric differences in the microstructure in terms of porosity, length and thickness of the trabecular system (figure 4).

Stiffness characterization highlighted the different elastic behaviour of these microstructures. In particular, all Young’s moduli have similar values for all RVEs of stereom 4, showing its almost isotropic behaviour. The slight spherical shape of the 3D directional representation of the longitudinal stiffness of stereom 4 indicate the lack of any definite stiffness specialization of such microstructure. Its almost isotropic nature is further highlighted by the low variability among the shear stiffness values and Poisson’s ratios.

Conversely, the distinct values of the Young’s moduli $E_{11}$, $E_{22}$ and $E_{33}$ of RVEs 1, 3 and 4 of stereom 5, demonstrate its anisotropic nature. The sole exception is represented by RVE 2, which exhibits a less anisotropic behaviour, yet presenting a value of $E_{11}$ that is about 8% higher than $E_{22}\approx E_{33}$.

The shear moduli and Poisson’s ratios of stereom 4 confirm its nearly isotropic behaviour. By contrast, stereom 5 displays higher shear stiffness in the ${\text{n}}_{2}-{\text{n}}_{3}$ plane and different Poisson’s ratios as $\nu_{32}$ and $\nu_{31}$ are lower than the others. However, RVE 2 of stereom 5 exhibits lower stiffness specialization.

Noticeably, stereom 5 revealed a non-homogeneous microstructure since RVE 2 results almost isotropic, while RVEs 1, 3 and 4 are clearly anisotropic. This is probably due to the spatial variability of the stereom. A further 3D inspection reveals the presence of distinct galleries departing from the centre of the plate towards the suture edges, with an angled orientation, as clearly shown in figure 9D. This characterizes the entire region attributed to stereom 5, which is highlighted by a yellow circle in this figure. As a representative example, in figure 9D is shown a cross section of RVE 1 of stereom 5 with the plane defined by y and $n_{1}$, that is the axis of the maximum longitudinal stiffness. This view of the microstructure exposes the galleried microstructure parallel to ${\text{n}}_{1}$.

Consequently, the stereom 5 could be classified as an inclined galleried stereom and, eventually, the plate centre could be considered a region of transition between a labyrinthic, isotropic microstructure such as RVE 2 of stereom 5, that gradually specializes to an inclined galleried microstructure, such as RVEs 1, 3 and 4 of stereom 5.

### 4.4. Stereom 6: the perforate high-density stereom at the plate basal zone

Stereom 6 corresponds to the high density microstructure characterizing the basal plate zone, consisting of a trabecular system with a low number of elements per unit volume and long and thick trabeculae (figure 4). As shown in figure 9A, stereom 6 has a distinctive dense structure that appears to be divided into two different geometric layers: a palisade-like layer at the top (p1) and a tortuous layer at the bottom (p2). Smith [14] classified these layers as a perforated over a labyrithic microstructure. Nonetheless, this labyrinth is noticeably different from the one located at the tubercle centre (stereom 4).

The stiffness tensor component results showed that this stereom is characterized by a relatively high stiffness since the Young’s moduli of all RVEs have higher values than those of all other stereoms, except $E_{11}$ of stereom 1. The slightly different values of the Young’s moduli along the three principal material directions showed the slight stiffness specialization of this microstructure. This is confirmed by the low variability of shear stiffness and Poisson’s ratios and by the slightly elongated surface representing the directional dependence of longitudinal stiffness (see, e.g. figure 8E). Notably, RVE 4 exhibited a higher stiffness specialization compared with the other RVEs since this particular sample is characterized by a value of $E_{11}$ that is about 40% higher than $E_{33}$ and 13% higher than $E_{22}$. However, the values of the Young’s moduli of such RVE are sensibly lower than the corresponding ones of RVEs 1, 2 and 3. A similar trend is exhibited by the shear stiffnesses, among which $G_{23}$ is larger than the others. A similar pattern emerges for the Poisson’s ratios, among which $\nu_{32}$, $\nu_{31}$, and $\nu_{21}$ have lower values with respect to the others. Such difference in stiffness specialization does not correspond to a significant variation in the orientation of the axes of maximum and minimum longitudinal stiffness. Actually, the direction of maximum longitudinal stiffness, namely $n_{1}$, is parallel to y for all RVEs, while the minimum longitudinal stiffness $n_{3}$ typically aligns with the z−x plane, albeit with slight variations in orientation among the four RVEs. The distinct orientation of $n_{3}$ is probably associated with the location of each specific RVE within the plate.

Interestingly, the orientation of the axes of maximum longitudinal stiffness, always parallel to y, appears to contrast with the orientation of the palisade-like structures mentioned earlier, which align along the z-axis.

### 4.5. The overall skeletal model of the *P. lividus* plate overall skeletal model of the *P. lividus* plate

**Figure 10.**
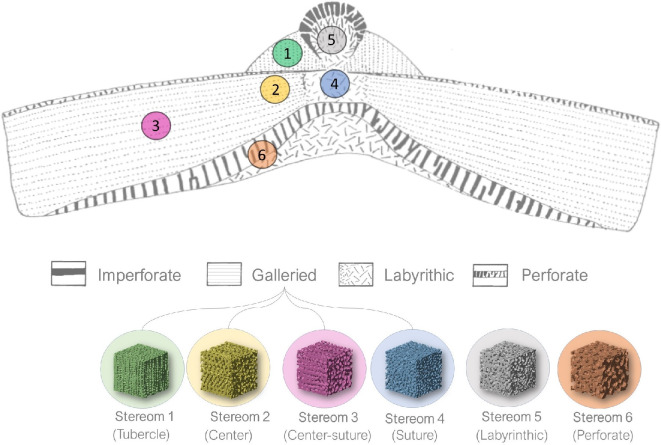
Stereom organization in the species-specific pattern of *P. lividus* and its comparison with previous literature [14]. The main stereom types analysed are illustrated, emphasizing key differences respect to Smith's model, including the distinction between the galleried stereoms 2 and 3, as well as the identification of stereom 5 as an angled galleried stereom, previously identified as labyrinthic.

The results of this study enabled the identification and characterization of the unique 3D variability of the echinoid stereom in terms of trabecular configuration, fabric anisotropy and stiffness properties. Comparing these results with previous 2D investigations [14,47] revealed additional key structural and mechanical differences, refining the understanding of the microarchitectural variability in the *P. lividus* inter-ambulacral plates (figure 10).

In 1980, Smith conducted a visual classification of the stereom micro-architecture within *P. lividus* plates, identifying the following structural bands (figure 10): a galleried stereom located at tubercle boss and sutures, a labyrinthic stereom at the centre of the tubercle and plate and a perforated stereom at the basal zone [14].

In this study, the results show a significant structural and mechanical distinction between the galleried stereom 1 (at the tubercle boss), stereom 2 (near the plate centre) and stereom 3 (closer to the plate edges), which was previously described as a single microstructure [14]. Additionally, stereom 5 located at the plate centre beneath the tubercle, is identified as an inclined galleried microstructure pertaining to a region of transition between the labyrinthic and the galleried microstructures (figure 10).

The mechanical properties derived from the 3D analysis also reveal key differences with respect to the previous 2D study [47]. For example, the galleried stereom described in the 2D investigations appears more rigid than the corresponding 3D volumes. Hence, even if the 2D analysis can provide some structural aspects, a 3D investigation is necessary to understand the effective elastic behaviour of these microarchitectures. As in the 2D description [47], these results align with the idea that the material properties and 3D architecture of the stereom are adapted to specific mechanical needs, as hypothesized in previous studies on echinoid plates and other skeletal elements [12,14,28,47,69,70]. Interestingly, the achieved results on the elastic constants not only provide useful insights about the anisotropic or isotropic mechanical behaviour of the stereoms, but also offer important information concerning the nature and direction of the forces operating on them and the entire plate.

Specifically, the orientations of the axes of maximum and minimum longitudinal stereomic stiffness support the assumption that the microstructure variation is probably related to the principal stress directions coming from the action of the primary spine, along with the direction of the collagen fibres and their stereomic insertion. As established in the theory of elastic plates (see, e.g. [71]) a plate supported at the edges and loaded by a concentrated force at the centre experiences principal stress directions radiating outward from the centre towards the edges. Accordingly, the stereom is characterized by high density imperforated microstructure at the tubercle top, which is probably subjected to stress concentrations induced by the spine action. At the tubercle boss, the stereom is specialized in a high-anisotropic galleried configuration, which is cable to transfer directional stresses of the catch apparatus fibres inserted along the galleries connecting the spine with the tubercle.

The centre of the tubercle presents a less stiff, isotropic labyrinthic stereom with non-oriented trabeculae. Its presence is probably due to lower stress values associated with a more isotropic stress state, where principal stress directions lack a predominant alignment. This hypothesis is further supported by the higher porosity of this region, which leads to reduced stiffness at the boss centre.

Below the tubercle, a transition region connects the labyrinthic microstructure to anisotropic galleried stereoms, which, with varying orientations, extend outward from the centre and directionally distribute applied stresses towards the suture areas. In these regions, the stresses align parallel to the plate mid-plane. The sutures are characterized by skeletal protrusions and collagen fibres, which reveal a compliant behaviour that may contribute to the dissipation of these directional stresses other than reducing the bending moments in the entire echinoid test [10].

Finally, a thick and stiff perforate stereom is present in the basal region, indicating that this area experiences stress concentrations.

## 5. Conclusion and future outlook

In nature, forms and structures generally adapt to a minimum use of energy and material for their construction and maintenance, as well as to ensure sufficient resistance to withstand mechanical forces. The study carried out in this paper seems to confirm this adaptation in the echinoid skeleton by revealing the unique specialization of its microarchitecture. The possible stereomic architectural modulations in relation to specific mechanical needs have been hypothesized and described in several studies regarding echinoid plates and other skeletal elements [12,14,28,69,72,73] as well as in other echinoderms, such as starfish [32].

Contemporary research into stereom architectures continues to reveal new 3D geometrical and compositional insights leading to superior mechanical performance, especially damage-tolerance [32,33]. Hence, the stereom seems to be a major evolutionary innovation in this phylum, combining minimal mass and strength to adapt to both predation and mechanical stresses in the marine environment.

The results of this study, presenting the first 3D geometrical description, fabric analysis and stiffness characterization of all stereom types in the *P. lividus* plate, represent a novel knowledge contribution, revisiting over 44 years of existing literature and provide useful foundations for future research in biomechanics and biomimetics.

Several aspects of the echinoid stereom can be further investigated including the intra- and interspecific geometric and mechanical analysis of the entire plate, the analysis of the transitional zones within the different stereoms, as well as fracture behaviour for each stereom type and the relation between the microarchitectural variability and applied static and dynamic loading conditions.

It is important to consider that the material composition of the calcite plays a fundamental role in influencing the structural performances and numerous studies reported unique mechanical effects, such as how the intercalation of Mg into biomineral structures improves the hardness [74–76] or how the co-alignment of the anisotropic calcite crystals with the microstructure leads to a more uniform stress distribution compared with isotropic material properties [32]. Accordingly, future studies can investigate in detail the material properties, compositional and crystallographic characterizations of the different stereom types and how they can influence the overall mechanical response.

Another fascinating area for further investigation is the role of organic components in stereom structures. Numerous studies have highlighted how the interplay between inorganic and organic interfaces in biological materials, such as nacre and bones, combined with their unique mineral architectures, enables nonlinear deformation processes [6,7,77,78]. This synergy transforms inherently brittle materials into ones capable of inelastic deformation, allowing them to redistribute stresses, absorb energy, and redirect cracks, enhancing the overall material’s toughness [6,7,77,78]. Hence, the consideration of organic components can surely add new predictions about mechanical properties of the different stereom types, potentially enhancing our understanding of their toughness, resilience and the ability to absorb energy during impacts.

All these aspects are objects of current study by the authors to better understand the meaning of this unique assemblage of anisotropic and isotropic trabecular geometries, also in terms of order and disorder. Ji *et al.* [45] have reported the high fracture resistance and energy dissipation effects of the echinoid-ordered stereom structures. Conversely, a recent work revealed that a certain degree of disorder observed in numerous natural cellular materials can also lead to improved damage tolerance with respect to fully ordered ones [11].

Echinoids seem to effectively combine both, as also reported in other studies [31]. This combination presumably enables a dynamic adaptation over space and time of skeletal trabecular templates and its material property distribution to the expected applied forces.

Understanding this type of strategy in echinoids as well as in the entire Echinodermata phylum can provide interesting suggestions for the development of graded porous ceramics with superior mechanical performances. Bioinspired porous structures are of high interest in several fields, such as aerospace, automotive and construction, where high-performance yet lightweight materials are essential [79–83].

The graded stereomic architecture, transitioning from isotropic to anisotropic regions, can provide a blueprint for the creation of functionally graded cellular materials with tunable stiffness, strength and energy absorption. This architectural principle has been extensively explored in bioinspired materials research, particularly in the development of engineered porous structures. As also effectively demonstrated in the literature, complex geometries of such materials can be digitally modelled and fabricated using algorithms such as Voronoi tessellation [84] or spinodal architected topologies (e.g. [83,85,86]). Notably, Kumar *et al.* [85] proposed a class of spinodal topologies that approximate microstructures observed during spinodal phase separation, providing a biomimetic approach for enhancing mechanical efficiency. Senhora [85] further analysed these structures, demonstrating their potential in 3D-printed applications by evaluating their mechanical properties. Xiang *et al.* [86] introduced a systematic design strategy for implementing spinodal materials to achieve customized mechanical responses. Furthermore, Desole *et al.* [87] investigated the energy absorption capabilities of PLA-based metamaterials incorporating spinodal structures, while Zheng *et al.* [88] further expands on their structural and functional applications.

As a future vision and innovation, lessons derived from the echinoid can be abstracted and translated into new inspired designs that are increasingly similar to the construction law of these organisms. The morphogenetic logic of the echinoid skeleton can be translated into a computational flow design in which the choice between isotropic and anisotropic architecture, as well as its composition, depends on the specific constraint of the materials, structure, shape and applied loads.

This vision can be thoroughly applied thanks to the digital revolution that now enables new design spaces and fabrication with a free formal expression. Digital modelling, analysis and fabrication are now dissolving the dichotomy between form and matter allowing the development and controlled deposition of complex material with continuous hierarchical shapes and multiscale structures. This empower a new vision in which the ‘learning from nature’ approach combined with nature-based materials and a coherent use of computational design and fabrication can be configured as a future line of human design able to imitate and integrate nature through multiple dimensions.

## Data Availability

Data have been provided in the electronic supplementary material [89].
